# Explainable AI-driven model for gastrointestinal cancer classification

**DOI:** 10.3389/fmed.2024.1349373

**Published:** 2024-04-15

**Authors:** Faisal Binzagr

**Affiliations:** Department of Computer Science, King Abdulaziz University, Rabigh, Saudi Arabia

**Keywords:** gastrointestinal cancer, explainable AI, SHAP, transfer learning, ensemble learning

## Abstract

Although the detection procedure has been shown to be highly effective, there are several obstacles to overcome in the usage of AI-assisted cancer cell detection in clinical settings. These issues stem mostly from the failure to identify the underlying processes. Because AI-assisted diagnosis does not offer a clear decision-making process, doctors are dubious about it. In this instance, the advent of Explainable Artificial Intelligence (XAI), which offers explanations for prediction models, solves the AI black box issue. The SHapley Additive exPlanations (SHAP) approach, which results in the interpretation of model predictions, is the main emphasis of this work. The intermediate layer in this study was a hybrid model made up of three Convolutional Neural Networks (CNNs) (InceptionV3, InceptionResNetV2, and VGG16) that combined their predictions. The KvasirV2 dataset, which comprises pathological symptoms associated to cancer, was used to train the model. Our combined model yielded an accuracy of 93.17% and an F1 score of 97%. After training the combined model, we use SHAP to analyze images from these three groups to provide an explanation of the decision that affects the model prediction.

## 1 Introduction

**Figure 1:**
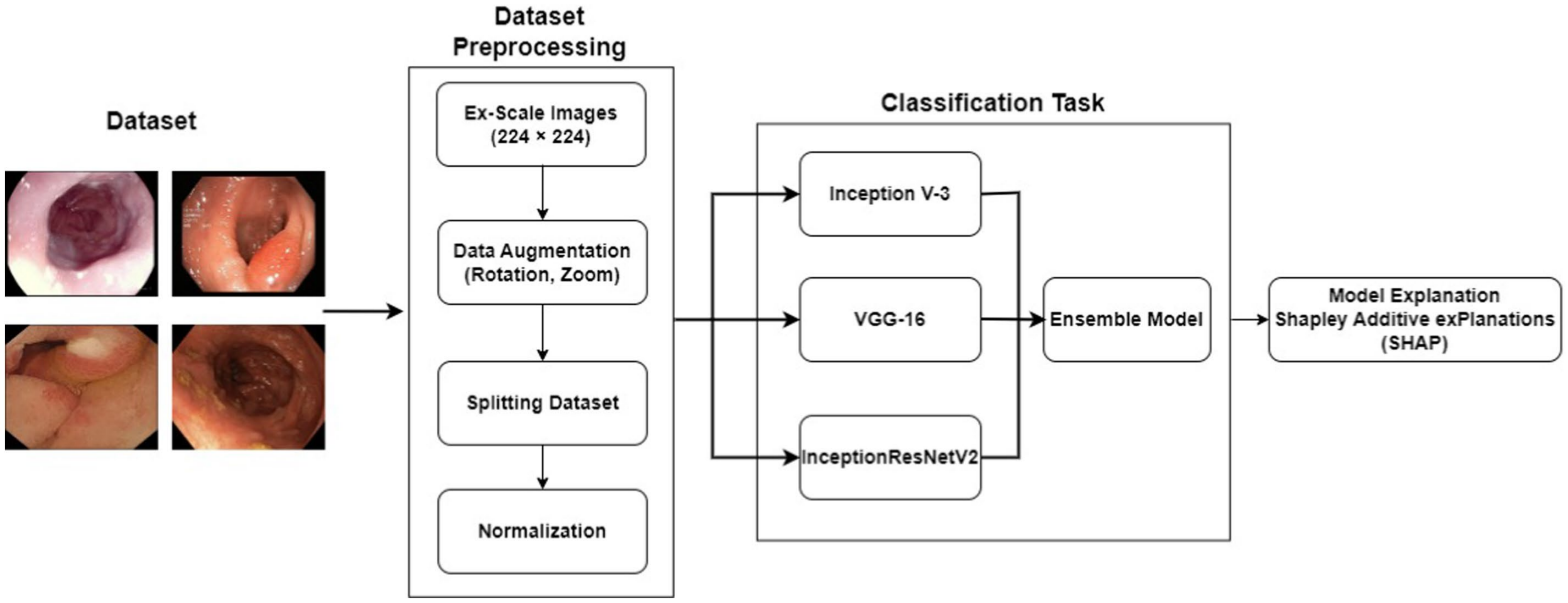
Proposed model.

The digestive system consists of the organs that make up the digestive system. Cellular mutations in at least one of these genes can lead to cancer and ultimately lead to the development of colon cancer. More importantly, colon cancer has a huge impact on the world, accounting for approximately 26.3% of all cancers (4.8 million cases) and 35.4% of blood cancer cases (3.4 million deaths) (1). As shown in Figure 1, the digestive system has a line that is approximately 25 feet long, starting from the mouth and ending at the anus. Many studies, including [Hospital] and (2), identified the most common types of cancer, including stomach, colon, colon, liver, and Cancer.

Latest studies have reported that a substantial proportion (over 50%) of gastrointestinal cancers can be attributed to risk factors that can be altered by adopting a healthier lifestyle alcohol intake, cigarette smoking, infection, unhealthy diet, and obesity (3, 4). Moreover, it has been observed that males have a higher susceptibility to gastrointestinal cancers compared to females, with the risk increasing with age, as indicated by (5). Unfortunately, due to late-stage diagnoses being predominant, the prognosis for such cancers is typically unfavorable (6), thus resulting in site-specific death rates that align with the incidence trends. However, if gastrointestinal cancers are detected in their early stages, the survival rate becomes higher in the five-year timeline (7). Nonetheless, a study conducted by (8) put forward that cognitive and technological issues contribute to significant diagnostic errors, despite the effectiveness of traditional screening procedures.

The Global-Cancer-Observatory (9) predicts a substantial increase in the global mortality and incidence rates of gastrointestinal (GI) cancers (10) by the year 2040. The mortality rate is projected to rise by 73%, reaching approximately 5.6 million cases, while the incidence rate is expected to increase by 58%, with an estimated 7.5 million new cases. These alarming statistics highlight the urgent need for the development of dependable systems to support medical facilities in obtaining accurate GI cancer diagnoses. Addressing this priority through innovative research endeavors becomes crucial to effectively combat the rising burden of GI cancers on a global scale.

Recent research has highlighted the potential of Artificial Intelligence (AI) in reducing misdiagnosis rates associated with conventional screening techniques, thereby enhancing overall diagnostic accuracy (11–23). The main reason for this accomplishment is the application of machines as well as deep-learning techniques. However, a significant hurdle faced by AI-supported systems is their perceived nature as computational” black boxes.” The lack of transparency in the decision-making processes of these AI models has resulted in hesitancy among healthcare institutions when it comes to adopting them for diagnostic purposes, despite their effectiveness (24–35). It is therefore important for AI researchers to integrate digestible explanations throughout the development of AI-aided medical applications, thus assuring healthcare practitioners while also clearing any doubts they might have.

In this context, XAI has emerged as a promising field that aims to address the computational difficulties posed by AI systems, warranting the provision of explanations for model predictions (36). By employing XAI techniques, AI researchers can enhance the interpretability and transparency of AI-driven diagnostic systems, thereby fostering trust and facilitating their integration into clinical practice. To address the challenges in AI driven diagnostic systems, this research work focuses on the investigation of SHAP. SHAP is an explanation approach for model predictions that was introduced by (37). In our study, we have utilized an ensemble model that we developed and trained on the pathology results obtained from the publicly accessible Kvasir dataset. By employing SHAP, we aim to provide interpretable explanations for the predictions made by our ensemble model, thereby enhancing the transparency and understandability of the AI-assisted diagnostic system.

To pinpoint the critical elements influencing the decision-making process, this study presents a unique approach for the categorization of gastrointestinal lesions. InceptionV3, Inception-ResNetV2, and VGG16 (Visual Geometry Group) architectures are used in the article to apply transfer learning. CNN Models are optimized for the goal of identifying gastrointestinal lesions like esophagitis, poylps and ulcerative-colitis by the application of enhancements and fine-tuning procedures. These improvements improve the precision and robustness of the models as compared with the latest techniques like (38–42). Creation of an Ensemble Model: By combining the predictions of every CNN model, the research project suggests and creates an ensemble model. By combining the advantages of several models, the ensemble model seeks to enhance classification performance by making use of the variety and complementary traits of its component models. The ensemble model is developed to classify gastrointestinal lesions in the dataset, and its performance is assessed. The study also thoroughly examines the characteristics that impact the classification procedure, illuminating the critical elements influencing precise lesion classification. Explainability (43, 44) characteristics made it possible to visualize the variables that contributed to each prediction in a comprehensible way, highlighting significant differences in performance that would not have been apparent otherwise.

The rest of the paper is organized as follows. Section II briefly gives a literature review; the framework of our novel technique is shown in Section III. Section IV presents experimental data, comments, and comparisons with existing techniques. Finally, the article is wrapped up in Section V.

## 2 Related work

Numerous research investigations have been carried out to develop automated models for detecting gastrointestinal cancer. According to (45), the detection of esophageal cancer using deep-learning (i.e., CNN) and machine-learning is becoming progressively prevalent. Preliminary screening of esophageal cancer has been made possible through the development of computer-assisted application by (46). Eventually, the researchers achieved the classification of esophageal images through the implementation of random forests as an ensemble classifier for esophageal image classification. Nonetheless, deep-learning models are being investigated.

In a study conducted in 2019 (47), developed a VGG16, InceptionV3, and ResNet50 model based on the transfer learning approach to classify endoscopic images into three classes: normal, benign-ulcer, and cancer using a custom dataset of 787 images including 367 samples of cancer, 200 samples of normal cases, and 220 samples of ulcers collected from Hospital. The images were first resized to 224×224 before they were preprocessed using Adaptive-Histogram-Equalization (AHE) to eliminate variations in the image brightness and contrast, thereby improving the local contrast and enhancing edge definition within each image region. Three binary classification tasks namely: normal vs. cancer, normal vs. ulcer, and cancer vs. ulcer were performed in this study and the accuracy, standard deviation, and Area-Under-Curve (AUC) values across the different CNN models. ResNet50 demonstrated the highest performance for all three-performance metrics. The model achieved an accuracy of above 92% for the classification tasks including the Normal images. However, for the cancer vs. ulcer task, a lower accuracy of 77.1% was noted. The authors conclude that this decrease is probably attributed due to the smaller visual differences between cancer and ulcer instances.

ResNet50 also achieved the lowest standard deviation, which indicates greater stability among the other models. In terms of AUC, ResNet50 reported an AUC of 0.97, 0.95, and 0.85, respectively for the normal vs. ulcer, normal vs. cancer, and cancer vs. ulcer tasks. The authors concluded that this proposed deep learning approach can be a valuable tool to complement traditional screening practices by medical practitioners thus reducing the risk of missing positive cases due to repetitive endoscopic frames or diminishing concentration.

The authors of (48) developed a deep CNN based on the UNet++ and Resnet50 architectures to classify between cases of gastritis (AG) and non-atrophic gastritis (non-AG) using white light endoscopy images. A total of 6,122 images (4,022 AG cases and 2,100 non-AG) were collected from 456 patients and were randomly partitioned into training (89%) and test sets (11%). For the binary classification task, the model achieved an accuracy of 83.70%, sensitivity of 83.77%, 13 and specificity of 83.75% while for the region segmentation task, an IOU score of 0.648 for the AG regions and 0.777 for the incisura region. The results suggest that the developed model based on the UNet++ and Resnet50 architectures can effectively distinguish between AG and non-AG cases, and it can also be used to delineate specific regions of interest within the endoscopic images.

Based on a research carried out by (49), images of non-cancerous lesions and Early-Gastric-Cancers (EGC) were used to evaluate CNN diagnostic potential. A dataset, comprising of 386 non-cancerous lesions images and 1702 ECG images, was used for the training of the CNN model. The analysis results showed a sensitivity level of 91.18% showing the model’s adeptness to rightly identify EGC cases and a specificity level of 90.64% indicating its ability to properly identify non-cancerous lesions. Substantially, reaching an accuracy level of 90.91% of the CNN model to diagnose both types of cases. Upon comparison, no remarkable differences were found between the specificity and accuracy levels of the AI-aided system and endoscopy specialists. However, the specificity and accuracy levels of the non-experts were below those of both the endoscopists and AI-aided system. According to the study findings, the CNN model exhibited exceptional EGC and non-cancerous lesions diagnostic performance. Consequently, this research demonstrates the potential of AI-aided systems in assisting medical practitioners.

In the study presented by (50), an automated detection approach utilizing Convolutional Neural Networks (CNN) was proposed to assist in the identification of Early-Gastric-Cancers (EGC) in endoscopic images. The method employed transfers learning on two distinct classes of image datasets: cancerous and normal. These datasets provided detailed information regarding the texture characteristics of the lesions and were obtained from a relatively limited data set. The CNN based network was trained using transfer learning techniques to leverage the knowledge acquired from pre-trained models. By utilizing this approach, the network achieved a notable accuracy of 87.6%. Subsequently, an external dataset was used for the evaluation of the model’s performance and an accuracy of 82.8% was attained.

The median filtering (MF) approach is used in the MSSADL-GITDC technique that is being presented to smooth images. The class attention layer (CAL) modifies the enhanced capsule network (CapsNet) model in the MSSADL-GITDC approach, which is offered for feature extraction. Deep Belief Network with Extreme Learning Machine (DBN-ELM) was utilized for GIT categorization. The accuracy of the suggested approaches was 98.03% (51). A unique approach for the automated identification and localization of gastrointestinal (GI) abnormalities in endoscopic video frame sequences is presented in this work (52). The photos used for training have poor annotations. The localization and anomaly detection performance obtained were both greater than 80% in terms of the area under the receiver operating characteristic (AUC).

These results suggest that the proposed automated detection method based on CNN, trained on the cancerous and normal image datasets, effectively aids in the identification of EGC in endoscopic images. The achieved accuracy of 87.6% on the training dataset demonstrates the model’s ability to discern between cancerous and normal instances. Furthermore, the comparable accuracy of 82.8% on the external dataset indicates the model’s generalizability and potential for practical application in clinical settings.

The idea of interpretable real-time deep-neural-network (SHAP) was first proposed by (53). The proposed technique showcased improved real time performance compared to existing methods. Experimental results highlighted the superiority of this approach over current deep learning techniques. Moreover, the author successfully addressed the needs of colorectal surgeons by providing satisfactory operational effectiveness and interpretable feedback. By incorporating Shapley additive explanations, the technique not only offers enhanced performance but also ensures interpretability, aligning with the requirements of the medical professionals in the field of colorectal surgery.

Upon investigation of prior research on the detection of gastrointestinal cancer using AI assistance, it became evident that this field will highly benefit from further exploration. While several AI models have been utilized to discover deformities in medical images, there remains a notable gap in the development of human-comprehensible models that can provide explanations for model predictions. Although there has been a recent surge of interest among researchers, only a limited number of studies have focused on creating AI models that offer interpretability, allowing healthcare professionals and stakeholders to understand and trust the predictions made by these models. In the context of gastrointestinal disease classification, the various shapes and sizes of a single lesion is a greater problem. Moreover, the single model extracts single type of features due to which classification accuracy reduces. Therefore, there is a clear need for more research efforts to develop AI models in gastrointestinal cancer detection that not only achieve high accuracy but also provide comprehensible explanations for their predictions.

## 3 Methodology

The proposed scheme jointly defines artificial intelligence (XAI) and presents an XAI-based model for gastrointestinal (GI) diagnosis. Figure 1 shows the proposed structure of XAI-based gastrointestinal cancer screening. The system was trained and evaluated using pathology results from the KvasirV2 dataset. A design was developed to improve the performance and accuracy of the system. This model incorporates predictions from multiple models and has the potential to increase power and improve overall classification. Additionally, the XAI process was used to uncover the determining factors associated with each category. This process can identify and describe key characteristics that influence the decision to classify the group. By integrating XAI into an integrated model and analyzing the decision, this approach aims to gain an understanding of the decision-making process of colon cancer testing, improving their transparency and interpretation.

### 3.1 Dataset

Datasets play a crucial role in the advancement of various computing domains, particularly in the field of deep learning applications. The availability and quality of datasets are critical since they must include enough examples, be sufficiently labeled, and show variety in the pictures. Several investigators and institutions have expanded the datasets for medical imaging so that it becomes easier to train and evaluate suggested models. This study made use of the Kvasir dataset initially introduced by (54) in 2017 which is composed of images that have been meticulously validated and annotated by medical experts. Each class contains a thousand images, thus showcasing pathological revelations, endoscopic approaches, and anatomical landmarks within the gastrointestinal tract.

However, for the purpose of this research, our focus was solely on the pathological findings class, which encompasses three distinct categories: Esophagitis, Polyps, and Ulcerative-Colitis, a chronic condition causing inflammation of the colon and rectum. To enhance the diversity and variety within the dataset, data augmentation techniques were applied to the original dataset. Specifically, rotation and zoom techniques were utilized to create variations of the existing images. This process involved rotating the images at different angles and applying zooming operations to produce new perspectives and scales.

**Figure 2:**
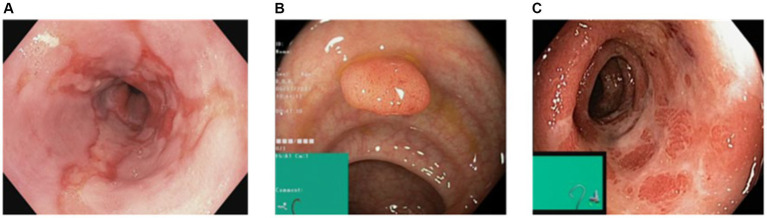
Sample images of dataset.

By applying these data augmentation techniques, another dataset having 2000 images per class was generated. This increased dataset size provided a broader range of image variations and ensured a more comprehensive representation of the pathological findings within the gastrointestinal (GI) tract. The augmented dataset with its increased variety and enlarged sample size is crucial for training and evaluating the proposed models effectively. It enables the models to learn from a more diverse set of examples and improves their ability to generalize and make accurate predictions on unseen data. Figure 2 shows the sample images from the dataset. The dataset was divided into training and testing ratios, with 70% data used for training and remaining 30% for testing.

### 3.2 Convolutional neural network models

There are a total of three primary deep CNN that were implemented in the development of the ensemble model. InceptionV3, created by (55) in 2015. InceptionV3 is an upgraded version of GoogleNet (Inception V1) and comprises 42 layers. VGG16, created by (56) in 2014, was the third model used to create the ensemble model. It has 16 layers and uses the Softmax classifier. Finally, the InceptionResNetV2 was effectuated. InceptionResNetV2 is deep-CNN having Inception-architecture as its foundational basis though it makes use of residual connections instead of undergoing the filter concatenation phase. It comprises of 164 layers and was formed in 2016 by (57).

### 3.3 Ensemble learning

Ensemble models are a valuable technique in machine learning that combines multiple individual models to enhance the overall performance of a system. The fundamental concept behind ensemble modeling is to leverage the strengths of different models to compensate for their respective weaknesses, resulting in improved accuracy, robustness, and generalization capabilities. Ensemble models come in several varieties, such as bagging, boosting, and stacking.

Several models are separately trained on various subsets of the training data in bagging. Usually, the final forecast is determined by combining all the models’ predictions together using methods like majority voting or averaging. This method aids in lowering overfitting and boosting forecast stability. Boosting, on the other hand, entails repeated training models. Each new model focuses on the examples that were misclassified by the previous models, thereby progressively improving the overall performance. Boosting algorithms assign higher weights to difficult examples, allowing subsequent models to prioritize those instances during training. Stacking takes a different approach by utilizing the predictions of multiple models as input features for a meta-model. The meta-model is trained to learn how to combine these predictions effectively and make the final prediction. This approach can capture complex relationships between the base models’ outputs and potentially improve overall performance.

Ensemble models find applications in various domains of AI, including computer vision, natural language processing, and speech recognition. For instance, in image classification tasks, an ensemble of CNNs can be employed to enhance accuracy and robustness. Each CNN within the ensemble may specialize in different aspects of feature extraction or classification, leading to improved classification performance. Ensemble models are a powerful technique in machine learning that leverages the collective wisdom of multiple models. By combining diverse models, ensemble methods can mitigate individual model limitations and yield superior performance across a range of AI applications (58–60). The mathematical equations behind the ensemble modeling is as follows:


$$\bar{f}\;\left(y|x\right)=\overset{T}{\underset{t=1}{\sum }}w_{t}\;f_{t}\left(y|x\right)$$

$$\bar{f}\;\left(y|x\right)=\frac{1}{Z}\;\overset{T}{\underset{t=1}{\prod }}f_{t}{\left(y|x\right)}^{w_{t}}$$

$$H\;\left(x\right)=\mathrm{sign}\;\left(\overset{T}{\underset{t=1}{\sum }}w_{t}\;h_{t}\left(x\right)\right)$$

Where a model f_t_ (y | x) is a model, moreover, and y is the estimated probability. Z is a normalization rule, h_t_ is a model output variable.

**Figure 3:**
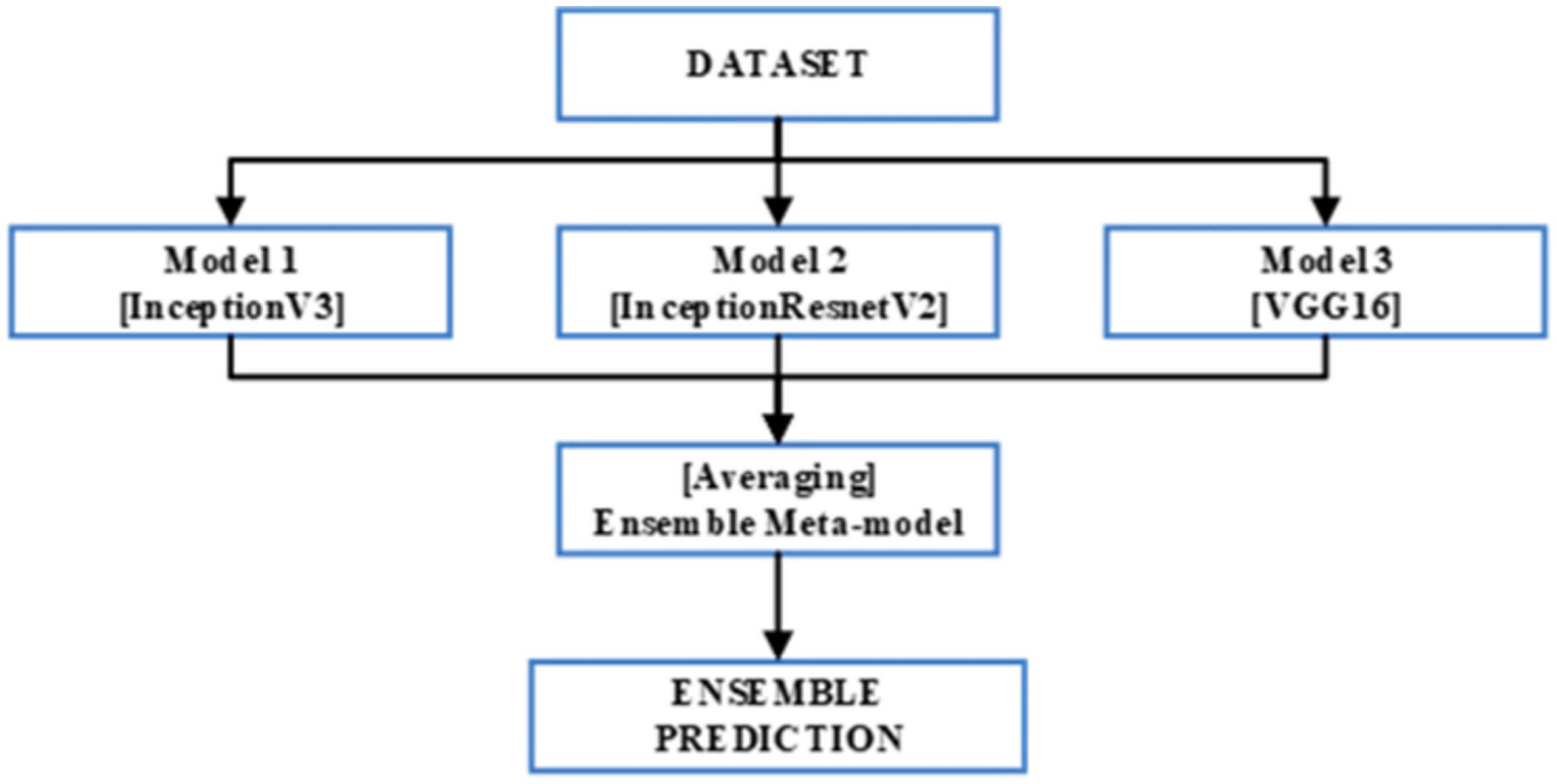
Ensemble model architecture.

This study focuses on the development of an ensemble model based on bagging techniques. This is executed through the synthesis of the predictions of three pretrained CNNs InceptionV3, InceptionResNetV2, and VGG16. The ensemble model’s architecture is depicted in Figure 3. Moreover, each of the three previously mentioned CNN models was applied to our enhanced KvasirV2 dataset which was separated into two parts 75% training and 25% validation. After individual training of the models, the average approach was used to develop the ensemble model through the combination of each model’s predictions. The average technique formulates an average of the predictions obtained from the three trained models resulting in the generation of the final prediction.

### 3.4 Explainable AI

The field of Explainable AI (XAI) is experiencing rapid growth, focusing on enhancing transparency and interpretability in machine learning algorithms. This advancement is of particular significance in the realm of medical imaging, as the outputs of machine learning models greatly influence patient care. XAI methods play a crucial role in enabling clinicians and radiologists to comprehend the rationale behind the model’s predictions, thereby instilling confidence in the accuracy of the model’s assessments. Moreover, XAI techniques aid in the identification of potential biases within the model, facilitating the prevention of misdiagnosis and promoting equitable healthcare outcomes (24, 25, 61).

XAI in medicine and healthcare have been classified in five categories by (36). Our goal is to improve the explain ability of medical imaging, which motivated us to investigate the XAI explanation using feature relevance approach. One such technique is the SHAP. The SHAP method developed by (37) is a model agnostic technique derived from cooperative game theory, enabling the interpretation of machine learning model outputs by quantifying the contribution of each feature. It provides a comprehensive framework that considers both global and local feature importance, accounting for feature interactions and ensuring fairness in assigning importance. The SHAP values align with desired axioms of feature attribution methods, including local accuracy, consistency, and missingness. Local accuracy ensures that the sum of SHAP values corresponds to the discrepancy between the model’s prediction and the expected output for a specific input. Consistency guarantees that fixing a feature’s value will not decrease its associated SHAP value. Missingness implies that irrelevant features have SHAP values close to zero. When you take into account all the many ways that features might combine, the Shapley value of a feature value indicates how much that feature contributed to the outcome (such as a reward or payout). It’s similar to calculating the relative contribution of each feature to the final result, accounting for all the various ways in which they may have cooperated. This aids in our comprehension of the elements that were crucial in reaching the desired outcome or making the final decision. The mathematical modeling for XAI is as:


$$g\;\left(\overset{´}{s}\right)=\varphi_{o}+\overset{M}{\underset{N=1}{\sum }}\varphi_{N}{\overset{´}{s}}_{N}$$

Here, $g\;\left(\overset{´}{s}\right)$ is the XAI model, $\overset{´}{s}$ is the simplified input such that $\left(\overset{´}{s}\right)$ ≈ (𝑥′) and $\overset{´}{s}$∈ (62) 𝑀 𝜙_𝑖_ ∈ 𝑅. Moreover, the function is defined in the manner shown in following equation to determine the impact of each attribute on model prediction.


$$\varphi_{N}=\frac{\left|∁\right|!\rho \left(\left|\rho \right|-∁-1\right)!}{\left|\rho \right|!}\;\left[\rho_{∁u\left\{N\right\}}\left(\sigma_{∁u\left\{N\right\}}-\rho_{∁}\left(X_{∁}\right)\right)\right]$$

Here, ∁ represents feature-sets, and ρ is a subset of ∁. $\rho_{∁u\left\{N\right\}}$ is the trained model on ∁ and N^th^ feature. $\rho_{∁}$ is the trained model without this feature. Moreover, $X_{∁}$ represent the feature-value in ∁^th^ set.

Various applications have benefited from SHAP values, encompassing domains such as image recognition, natural language processing, and healthcare. For instance, in a study focusing on breast cancer detection, SHAP values were utilized to identify the most relevant regions in the images (63). Similarly, in another study concerning the detection of relevant regions in retinal images for predicting disease severity (64), SHAP values were employed to interpret the features of a deep neural network model.

## 4 Experimental results

In the initial phase of the experiments, an ensemble model was developed for the classification of gastrointestinal (GI) lesions. This involved training the InceptionV3, InceptionResNetV2, and VGG16 models individually on the KvasirV2 datasets. Subsequently, these models were combined to create the ensemble meta model. To adapt the primordial architectures of the 3 pre trained convolutional neural networks (CNNs), a global average pooling layer was added. This layer summarizes the spatial information from the previous layers and reduces the dimensionality of the feature maps. Following the pooling layer, a dropout layer with a dropout rate of 0.3 was applied to mitigate overfitting, enhancing the model’s generalization capabilities. The Adam optimization algorithm was employed for model optimization, using sparse-categorical-cross entropy as the loss-function. This combination allowed for effective training of the ensemble model by updating the model weights based on the calculated gradients. Each of the selected deep-CNNs was trained for 5 epochs, with a batch size of 32, to finetune the model parameters and improve its performance. After the training process, the softmax activation function was utilized for classification. This function assigned probabilities to each class, enabling the ensemble model to make predictions on the GI lesion classes.

**Figure 4:**
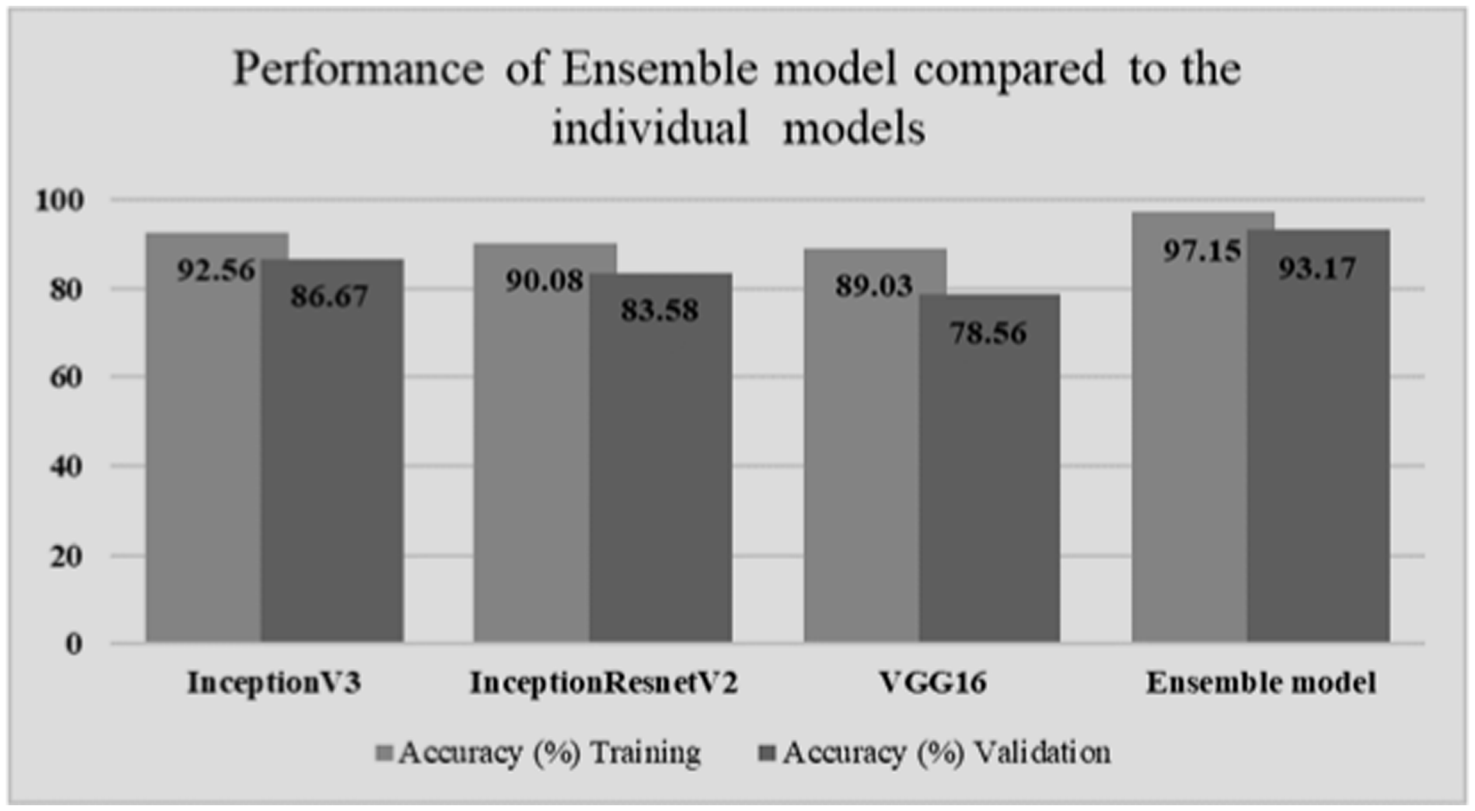
Classification comparison among models.

By leveraging the strengths of multiple pre-trained CNN models through ensemble learning, the developed model aimed to enhance the accuracy and robustness of GI lesion classification. Table 1 shows the classification results obtained on individual models as well as Ensemble model. Moreover, for comparison among individual models as well as ensemble model, a graph is shown in Figure 4.

**Table 1:** Classification performance on individual models.

Model	Training accuracy (%)	Validation accuracy (%)
Inception V3	92.56%	86.67%
InceptionResnetV2	90.08%	83.58%
VGG16	89.03%	78.56%
Ensemble model	97.15%	93.17%

**Figure 5:**
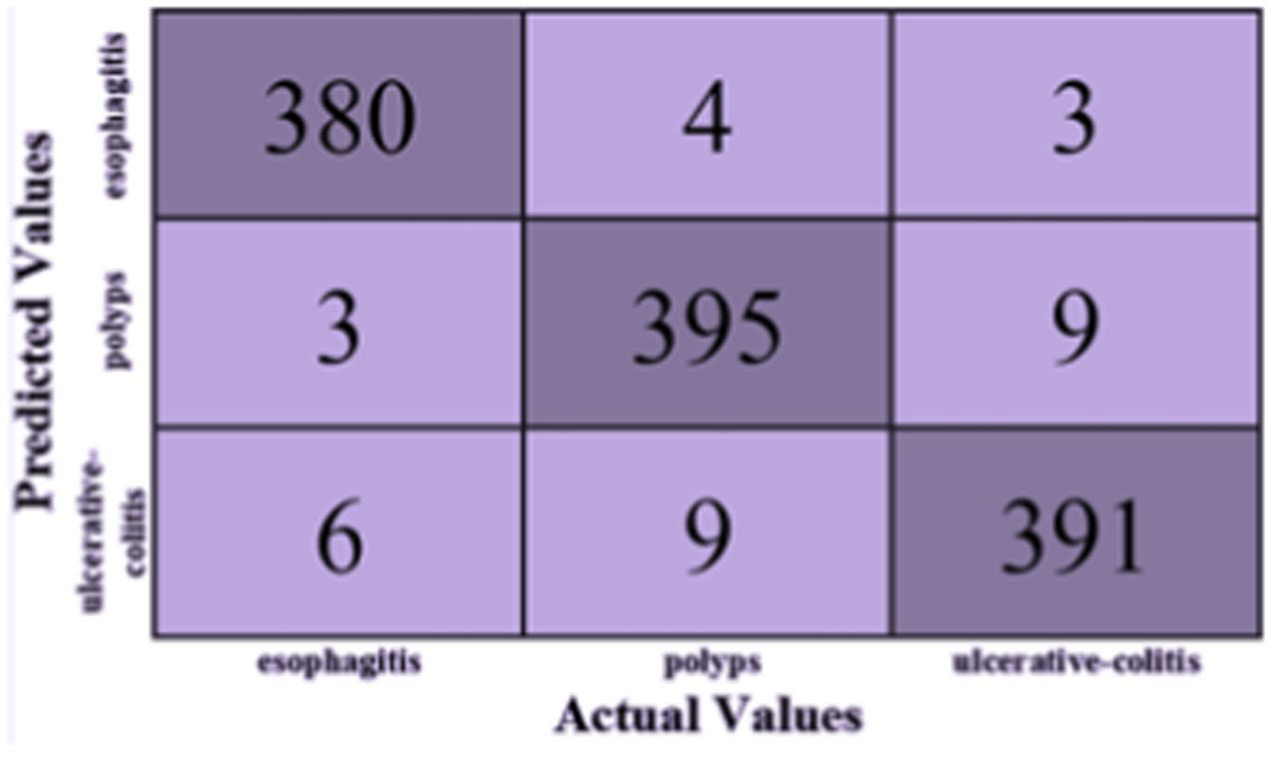
Confusion matrix of proposed model.

**Figure 6:**
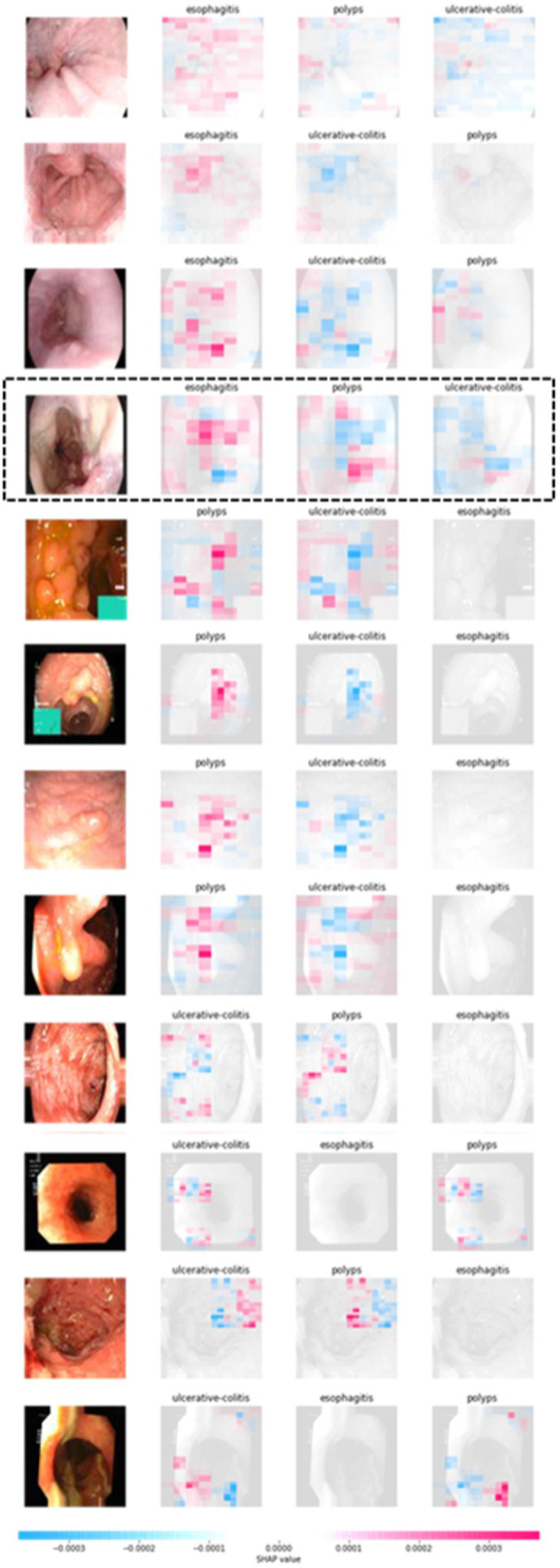
SHAP XAI explanation.

Based on Figure 5, the F1-score, recall, and precision for each class of esophagitis, polyps, and ulcerative colitis are displayed in the classification report, as shown in Figure 6. The confusion matrix derived from the classification results is shown in Figure 5. The confusion matrix, which displays the proportion of properly and erroneously identified samples for each class, offers a summary of the model’s performance.

The classification report is shown in Table 2 and contains the F1-score, recall, and accuracy metrics for the ulcerative colitis, polyps, and esophagitis classes. The model’s accuracy is gaged by the F1-score, which unifies recall and precision into a single number. The capacity of the model to accurately identify positive samples is reflected in recall, and the ability to correctly categorize positive predictions is reflected in accuracy. These metrics shed light on how well the model performs for a given class. The classification report and the confusion matrix provide useful data to assess the precision and potency of the created ensemble model in categorizing gastrointestinal lesions.

**Table 2:** Classification performance of ensemble model using various metrics on individual class.

Class/Label	Precision	Recall	F1-Score
Esophagitis	0.977	0.982	0.979
Polyps	0.968	0.971	0.969
Ulcerative-Colitis	0.970	0.963	0.967

When compared to the individual models, the ensemble model’s findings show a notable improvement in overall accuracy. The three classes—ulcerative colitis, polyps, and esophagitis—showcase excellent accuracy, recall, and F1-score in the classification report. These metrics show that the model can effectively minimize false positives and false negatives while correctly detecting positive events. An overall accuracy of 93.17% and an F1-score of 97% for every class show that the ensemble model performs well in classifying gastrointestinal lesions. The model can properly detect both positive and negative examples, as suggested by the high F1-score, which also indicates that the model strikes a balance between precision and recall. The encouraging outcomes of the ensemble model point to its potential for further refinement and implementation in clinical settings, which is appropriate considering the significance of precise prediction in the context of gastrointestinal malignancies. The model is a useful tool in healthcare practice as it may help diagnose GI cancer because of its high F1-scores and overall accuracy.

We employed a blurring-based masker in conjunction with the SHAP partition explainer to gain an understanding of the deterministic elements that underlie the predictions of our ensemble model. We were able to explain the accurate forecasts by using this method to visualize the precise regions of the image that were important to the model’s predictions. Four photos from each class—ulcerative colitis, polyps, and esophagitis—that our model properly predicted were included in this study. We were able to generate a visual depiction of each class’s contributing attributes by utilizing the SHAP partition explainer. The deterministic characteristics and their significance for the ulcerative colitis, polyps, and esophagitis groups are shown in Figure 6.

These visualizations improve the interpretability and explain the ability of our model’s predictions by offering insightful information about the areas or patterns within the pictures that had a major impact on the ensemble model’s decision-making process. The chart shows the real image in Figure 6, with blue and red highlights in particular areas. The red color indicates elements that positively added to the prediction of a particular category, while the blue color represents parts that had an adverse contribution. By analyzing the fourth image in Figure 6 as an example, it can be observed that the red shades are predominantly concentrated around the region corresponding to the esophagitis pathology in the esophagitis class.

**Figure 7:**
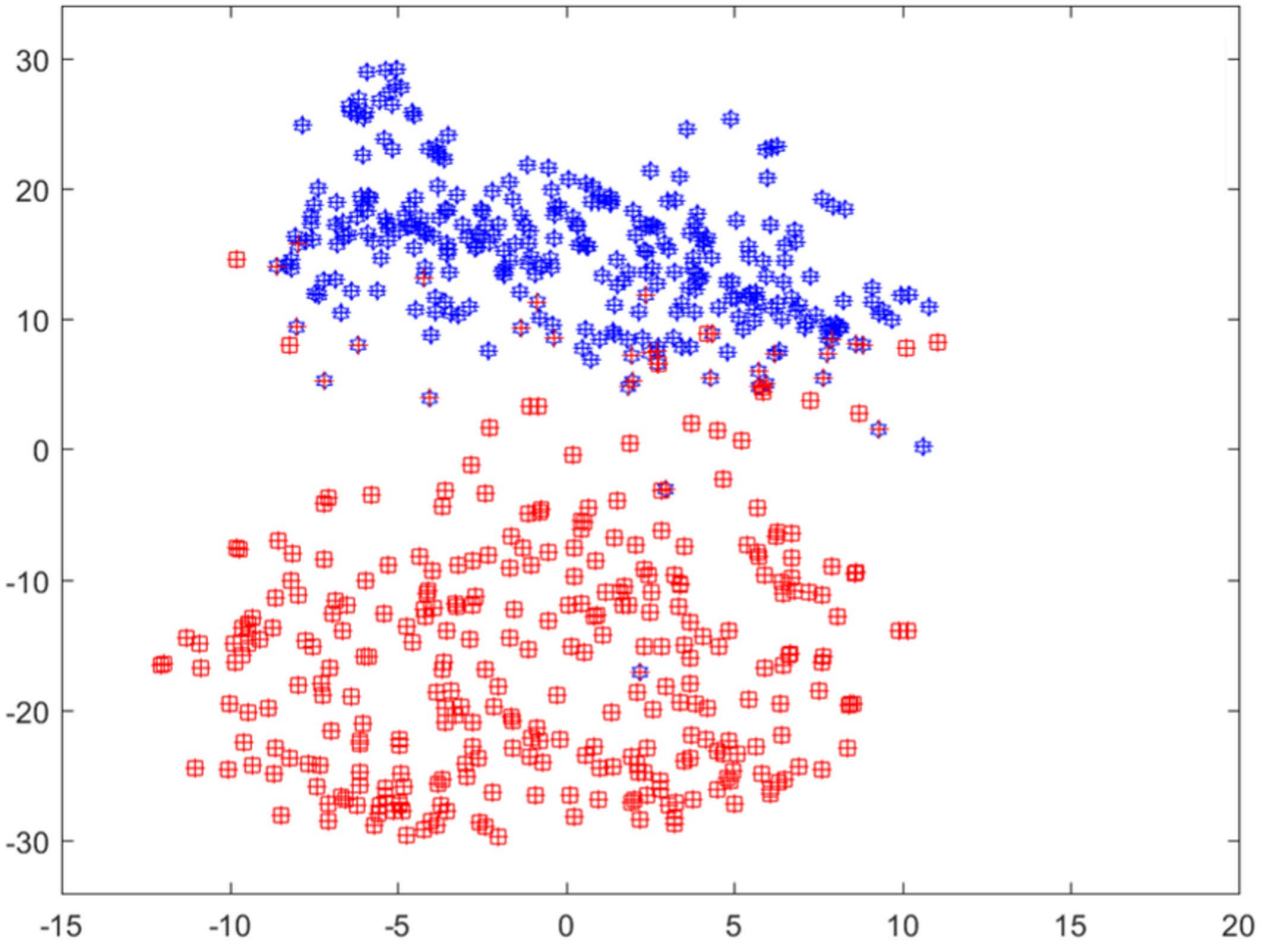
Features visualization using T-SNE.

This suggests that these highlighted regions played a significant role in the model’s prediction for this category. However, when examining the subsequent two classes predicted by the model, we notice that both images exhibit mostly blue shades in the area associated with esophagitis pathology. This implies that these regions negatively influenced the model’s prediction for these classes. Overall, the model predicts and outputs the deterministic features of each tested image, highlighting the regions that contribute positively or adversely to the predicted categories. This provides valuable insights into the specific image characteristics that the model considers when making its predictions. Moreover, features visualization using t-sne is also shown in Figure 7.

The limited number of studies conducted on gastrointestinal cancer detection highlights the need for further research in this area. Existing studies have reported moderate to high accuracies using deep learning models such as InceptionResNetV2 and InceptionV3. For instance, one study (65) achieved an accuracy of 84.5% using InceptionResNetV2 with a dataset of 854 images, while another study (49) reported an accuracy of 90.1% using InceptionV3 with a test set of 341 endoscopic images.

In comparison, our optimized ensemble model, along with the individual models, demonstrates superior performance compared to these existing studies. The accuracy of our ensemble model is reported as 93.17% with an F1-score of 97% for each class. This indicates the effectiveness of our approach in accurately classifying gastrointestinal lesions. However, it is important to acknowledge the challenges faced in developing and evaluating deep learning models for gastrointestinal cancer due to the limited availability of publicly accessible datasets in this domain. This scarcity hinders the progress and thorough evaluation of deep learning models for gastrointestinal cancer detection. Moreover, the lack of explainability in deep learning models has contributed to the hesitation among healthcare professionals in adopting these models in clinical practices. To address this limitation, our proposed model incorporates the SHAP technique, which allows for the identification of deterministic features within the images associated with gastrointestinal pathologies. By providing explanations for the model decision-making process, our model enhances the interpretability and trustworthiness of the results. It has been observed that major misclassification occurs in the ulcerative-colitis and then polyp class. This occurs as both have similarity in their shape and size, the problem can be catered by applying the contours and highlighting the region, which will be done in future work. The limitation of the model is its reproducibility of results which is generally a deep learning issue, moreover the technique is not evaluated on a real time system therefore it should be trialed clinically before implementation of it.

The comparison of the proposed model with the latest techniques is shown in Table 3.

**Table 3:** Performance comparison.

Techniques	Accuracy
AHE is applied on images after that ResNet50 is finetuned using transfer learning (47)	92.00%
Deep CNN based on the UNet++ and Resnet50 architectures to classify (48)	83.70%
Images of non-cancerous lesions and EGC were used to evaluate CNN diagnostic potential (49)	90.91%
Proposed approach	93.17%

## 5 Conclusion

The use of technology in healthcare is often challenged by a lack of explanation. This research addresses this issue by examining SHAP (Shapley Additive exPlanations) technology in depth. Colon cancer pathology results can be used to extract preliminary characteristics thanks to SHAP. The use of SHAP in our research aims to enhance the comprehension and interpretation of the prediction model. We start our study by creating and improving augmented ensemble models. The averaging approach was used to merge three pre-trained CNN models: InceptionV3, InceptionResNetV2, and VGG16. Pathology findings from the KvasirV2 dataset, a helpful tool for diagnosing gastrointestinal disorders, were analyzed for this sample. Co-learning maximizes the model’s quality by increasing the model’s accuracy and efficiency. Because a pooled sample incorporates the unique strengths and capacities of each sample, cancer detection using it can be more robust and trustworthy. Furthermore, each disease’s characteristic traits were highlighted using the SHAP translator method. With the use of this technology, we can decipher certain details and regions of medical pictures, enabling the creation of prediction models. We may gain a better grasp of the decision-making mechanism and the underlying concepts of forecasting by extracting and visualizing these elements. Our results demonstrate the acceleration, quality, and use of descriptive intelligence (XAI) models for cancer detection, particularly in colon cancer. For future work, we will investigate other AI models, explainability methodologies, or applicability to other forms of cancer or disorders.

## Data availability statement

The data that support the findings of this study are available from the first and corresponding authors upon reasonable request.

## Author contributions

FB: Conceptualization, Formal analysis, Funding acquisition, Investigation, Methodology, Project administration, Resources, Software, Validation, Visualization, Writing – original draft, Writing – review & editing.
